# Mechanism of inhibition of mouse Slo3 (K_Ca_5.1) potassium channels by quinine, quinidine and barium

**DOI:** 10.1111/bph.13214

**Published:** 2015-07-14

**Authors:** David C Wrighton, Stephen P Muench, Jonathan D Lippiat

**Affiliations:** School of Biomedical Sciences, Faculty of Biological Sciences, University of LeedsLeeds, UK

## Abstract

**Background and Purpose:**

The Slo3 (K_Ca_5.1) channel is a major component of mammalian KSper (sperm potassium conductance) channels and inhibition of these channels by quinine and barium alters sperm motility. The aim of this investigation was to determine the mechanism by which these drugs inhibit Slo3 channels.

**Experimental Approach:**

Mouse (m) Slo3 (K_Ca_5.1) channels or mutant forms were expressed in *Xenopus* oocytes and currents recorded with 2-electrode voltage-clamp. Gain-of-function mSlo3 mutations were used to explore the state-dependence of the inhibition. The interaction between quinidine and mSlo3 channels was modelled by *in silico* docking.

**Key Results:**

Several drugs known to block KSper also affected mSlo3 channels with similar levels of inhibition. The inhibition induced by extracellular barium was prevented by increasing the extracellular potassium concentration. R196Q and F304Y mutations in the mSlo3 voltage sensor and pore, respectively, both increased channel activity. The F304Y mutation did not alter the effects of barium, but increased the potency of inhibition by both quinine and quinidine approximately 10-fold; this effect was not observed with the R196Q mutation.

**Conclusions and Implications:**

Block of mSlo3 channels by quinine, quinidine and barium is not state-dependent. Barium inhibits mSlo3 outside the cell by interacting with the selectivity filter, whereas quinine and quinidine act from the inside, by binding in a hydrophobic pocket formed by the S6 segment of each subunit. Furthermore, we propose that the Slo3 channel activation gate lies deep within the pore between F304 in the S6 segment and the selectivity filter.

**Table:** Tables of Links

TARGETS	
KCa1.1 (Slo1; BKCa)	
KCa5.1 (Slo3)	

LIGANDS		
Ba2+ (BaCl)	Mibefradil	Quinidine
Clofilium	TEA+	Quinine

These Tables list key protein targets and ligands in this article which are hyperlinked to corresponding entries in http://www.guidetopharmacology.org, the common portal for data from the IUPHAR/BPS Guide to PHARMACOLOGY (Pawson *et al*., 2014) and are permanently archived in the Concise Guide to PHARMACOLOGY 2013/14 (Alexander *et al*., 2013)

## Introduction

A number of substances, including Ba^2+^ and quinine, affect spermatozoa motility and cell volume regulation by inhibiting K^+^ channels (Yeung and Cooper, 2001; Yeung *et al*., 2003; Barfield *et al*., 2005a,b[Bibr b3],[Bibr b4]). Slo3 (K_Ca_5.1) is a strong candidate for the predominant K^+^ channel in spermatozoa membranes, KSper (sperm potassium conductance) (Navarro *et al*., 2007; Santi *et al*., 2010; Zeng *et al*., 2011; 2013[Bibr b33],[Bibr b34]; Brenker *et al*., 2014; Mansell *et al*., 2014). It is also the closest molecular relative of the large-conductance Ca^2+^-activated K^+^ channel Slo1 (BK_Ca_, maxi-K, K_Ca_1.1), and is strongly expressed in testes (Schreiber *et al*., 1998). The mouse (m) Slo3 (K_Ca_5.1) channel is activated by increased cytosolic pH and not by intracellular Ca^2+^ (Schreiber *et al*., 1998; Zhang *et al*., 2006). In mouse spermatozoa, a Na^+^-dependent Cl^−^/HCO_3_^−^ exchanger is responsible for intracellular alkalinization during capacitation (Zeng *et al*., 1996), which is thought to activate KSper and hyperpolarize the cell membrane to more negative potentials. This could increase the driving force for Ca^2+^ influx through CatSper channels, thereby increasing sperm motility (Navarro *et al*., 2007), although an indirect link between Slo3 and CatSper function involving H^+^ transporters has also been proposed (Chavez *et al*., 2014). Male mice deficient in Slo3 K^+^ channel subunits (*Kcnu1* knockout) are infertile and have reduced alkalinization-induced K^+^ conductance (Santi *et al*., 2010; Zeng *et al*., 2011; 2013[Bibr b33],[Bibr b34]). In contrast, human spermatozoa undergo cytoplasmic alkalinization during capacitation as a result of proton transport by human voltage-gated proton channel activity (Lishko *et al*., 2010). Interestingly, the human Slo3 (K_Ca_5.1) channel has recently been shown to be activated by Ca^2+^ and less reliant on alkalinization, but still a critical component of KSper channels (Brenker *et al*., 2014). Given its importance in sperm physiology, human mutations in the gene encoding Slo3, *KCNU1*, are likely to be linked to male fertility. Hence, sperm ion channels are promising targets for drugs that either suppress or enhance male fertility (Lishko *et al*., 2012).

Despite the technical difficulties in recording KSper currents from spermatozoa and the poor heterologous expression of mSlo3 subunits, some of their functional and pharmacological properties have been correlated. Both KSper and mSlo3 are blocked by Ba^2+^, exhibit weak sensitivity to TEA^+^ relative to other K^+^ channels (Schreiber *et al*., 1998; Navarro *et al*., 2007; Martinez-Lopez *et al*., 2009), are enhanced by PKA (Martinez-Lopez *et al*., 2009), and inhibited by depletion of phosphatidylinositol 4,5-bisphosphate (Tang *et al*., 2010a). Native KSper channels are also inhibited by quinine, clofilium, mibefridil and 5-(N-ethyl-N-isopropyl)-amiloride (Navarro *et al*., 2007). Detailed aspects of the pharmacology of Slo3 channels, including their block by 4-aminopyridine and quinidine, have been investigated by generating and expressing an mSlo3 chimera, MC13, which incorporates transmembrane regions from Slo1 (K_Ca_1.1) channels to improve expression in *Xenopus* oocytes (Tang *et al*., 2010b).

The aim of this study was to investigate the effects of inhibitors on full-length mSlo3 channels expressed in *Xenopus* oocytes. We were particularly interested in the state dependence of block by quinine, quinidine and Ba^2+^, and in comparing their effects on those reported with native KSper and the MC13 Slo1/Slo3 chimera (Navarro *et al*., 2007; Tang *et al*., 2010b). To explore state dependence, we generated gain-of-function mutants based on mutations previously shown to increase the open probability of Slo1 channels: R196Q is equivalent to an S4 mutation that enhances voltage activation (Diaz *et al*., 1998), whereas F304Y is analogous to a pore mutation that directly increases open probability (Lippiat *et al*., 2000). We found that channel inhibition by these substances was not dependent on channel activity; however, the binding site for quinine and quinidine was affected by the F304Y mutation in the mSlo3 pore.

## Methods

### Molecular biology and oocyte injection

A plasmid containing the full-length mSlo3 sequence was a gift from Dr L. Salkoff (Washington University School of Medicine) and the open-reading frame was subcloned into pBF (Baukrowitz *et al*., 1999), a vector optimized for *in vitro* mRNA synthesis and *Xenopus* oocyte expression. This ion channel subunit also has the designation K_Ca_5.1 (Alexander *et al*., 2013), but is termed mSlo3 in this paper. The R196Q and F304Y mutations were introduced by Quikchange site-directed mutagenesis (Agilent Technologies, Stockport, UK) and confirmed by automated sequencing. *In vitro* transcription was carried out using the mMessage mMachine SP6 kit (Ambion, Huntingdon, UK) to produce capped mRNA. Individual oocytes were obtained from humanely killed female *Xenopus laevis* toads (housed either at the University of Leeds, UK or from the European *Xenopus* Resource Centre, University of Portsmouth, UK) and were injected with 10–50 ng RNA in a 40 nL volume. Oocytes were incubated for at least 2 days at 18^o^C in a modified Barth’s solution comprising, in mM, 84 NaCl, 1 KCl, 2.4 NaHCO_3_, 0.82 MgSO_4_, 0.41 CaCl_2_, 0.33 Ca(NO_3_)_2_, 5 HEPES, adjusted to pH 7.4 with NaOH and supplemented with 100 IU·mL^−1^ penicillin/streptomycin, 2 mM pyruvate and 50 mg·mL^−1^ gentamycin. For oocytes injected with mutant Slo3 mRNA, survival was improved by raising the KCl concentration of the Barth’s solution to 23 mM. This brought the resting membrane potential, estimated from the Goldman–Hodgkin–Katz equation, reported oocyte intracellular ion concentrations (Costa *et al*., 1989), and an increased K^+^ conductance, closer to −30 mV and within the range of that of a non-injected oocyte.

### Electrophysiology

Microelectrodes were pulled from GC100F borosilicate glass capillaries (Harvard Apparatus, Cambridge, UK) and had resistances of between 0.3 and 5 MΩ when filled with 3 M KCl. Oocytes were voltage-clamped by an Axon GeneClamp 500 amplifier (Molecular Devices, Sunnydale, CA, USA) and currents were digitized with a NI USB-6211 interface (National Instruments, Newbury, UK) and recorded using WinWCP v4.0.5 (Strathclyde Electrophysiology Software, University of Strathclyde, UK). To generate current–voltage relationships, oocytes were held at −80 mV and pulses from −100 to +140 mV were applied at 0.2 Hz. For studies of drug block, pulses were applied to +100 mV followed by a 1.5 s voltage ramp from −100 mV to +100 mV. Oocytes were perfused at room temperature (20–22°C) with a Ringer’s solution containing, in mM, 115 NaCl, 2.5 KCl, 10 HEPES, 1.8 CaCl_2_, pH 8 with NaOH. The volume of the bath was 0.5 mL, which was perfused at a rate of approximately 1 mL·min^−1^. 100 mM K^+^ solution was made by an equimolar replacement of NaCl with KCl.

### Homology modelling and drug docking

Two homology models for the mSlo3 transmembrane domains were generated using PHYRE2 (Kelley and Sternberg, 2009) and SWISS-MODEL (Arnold *et al*., 2006), using the coordinates of a prokaryotic cyclic nucleotide-gated K^+^ channel (PDB : 3BEH) as a template, and the tetrameric structural models were created by fitting the individual subunits into the tetrameric crystal structure (Clayton *et al*., 2008) using DeepView (Swiss Institute of Bioinformatics, Lausanne, Switzerland). The binding of quinidine to mSlo3 homology models was studied *in silico* with SwissDock (Grosdidier *et al*., 2011). Electrostatic calculations were performed by pdb2pqr (Dolinsky *et al*., 2004). Molecular models were visualized and presented using PyMol (Schrödinger LLC, New York, NY, USA).

### Materials

All chemicals were reagent grade and obtained from Sigma-Aldrich (Poole, UK), unless stated otherwise. Quinine (Acros Organics, Loughborough, UK), quinidine, mibefradil, clofilium, barium chloride and tetraethlyammonium chloride were dissolved in either DMSO or Ringers solution to generate stock solutions, and then diluted to the required concentrations in Ringers solution.

### Data analysis and statistical procedures

Data were analysed with WinWCP, OriginPro 7.5 (Originlab, Northampton, MA, USA), and GraphPad Prism 6 software (GraphPad Software, San Diego, CA, USA). For drug inhibition analysis, drug-containing solutions were perfused until there was no further change in current amplitudes (typically 3–5 min), which were measured towards the end of depolarizing steps to +100 mV. Current (I)-inhibition plots were fitted with the equation: I = (I_0_ − C)/(1 + ([B]/IC_50_)^nH^) + C where I_0_ is the current in the absence of inhibitor, [B] the inhibitor concentration, IC_50_ the concentration that achieves half-maximal inhibition, n_H_ the Hill coefficient, and C the drug-insensitive component of the current. To measure the voltage dependence of current inhibition by quinine, quinidine and Ba^2+^, current amplitudes evoked by a voltage ramp were measured at sections corresponding to different voltages. The IC_50_–voltage relationships were fitted with the Woodhull equation in the form ln(IC_50_) = ln(IC_50_0).e^−δzVF/RT^, where IC_50_0 is the apparent IC_50_ at 0 mV, δ the fraction of the voltage field sensed by the blocking ion, z is the valence of the blocking ion (+1 for quinine and quinidine, and +2 for Ba^2+^), and F, R and T have their standard thermodynamic meanings. Data are presented as the mean ± SEM of *n* individual oocytes and statistical significance tested using Student’s *t*-test, anova (with Bonferroni *post hoc* comparison of means), or by Kruskal–Wallis test (with Dunn’s *post hoc* comparison) as indicated in the Results.

## Results

### Expression of wild-type (WT) and mutant mSlo3 channels

Full-length WT, R196Q and F304Y mSlo3 K^+^ channel subunits were expressed in *Xenopus* oocytes and currents recorded by two-electrode voltage clamp. Oocytes injected with WT mSlo3 gave rise to outwardly rectifying currents, which were absent in non-injected oocytes in the 0–100 mV range (Figure 1A and B). Oocytes injected with mRNA encoding the mSlo3 mutants R196Q and F304Y also yielded large outwardly rectifying currents, but exhibited channel activity at voltages below the threshold for WT mSlo3 activation (Figure 1A and B). Expressing R196Q and F304Y mSlo3 resulted in a significantly more negative oocyte resting membrane potential (Figure 1C) compared with control oocytes or those expressing WT mSlo3. We observed a reduction in the survival of oocytes expressing the gain-of-function mutants in normal Barth’s medium, which was rectified by raising the K^+^ concentration (see Methods).

**Figure 1:**
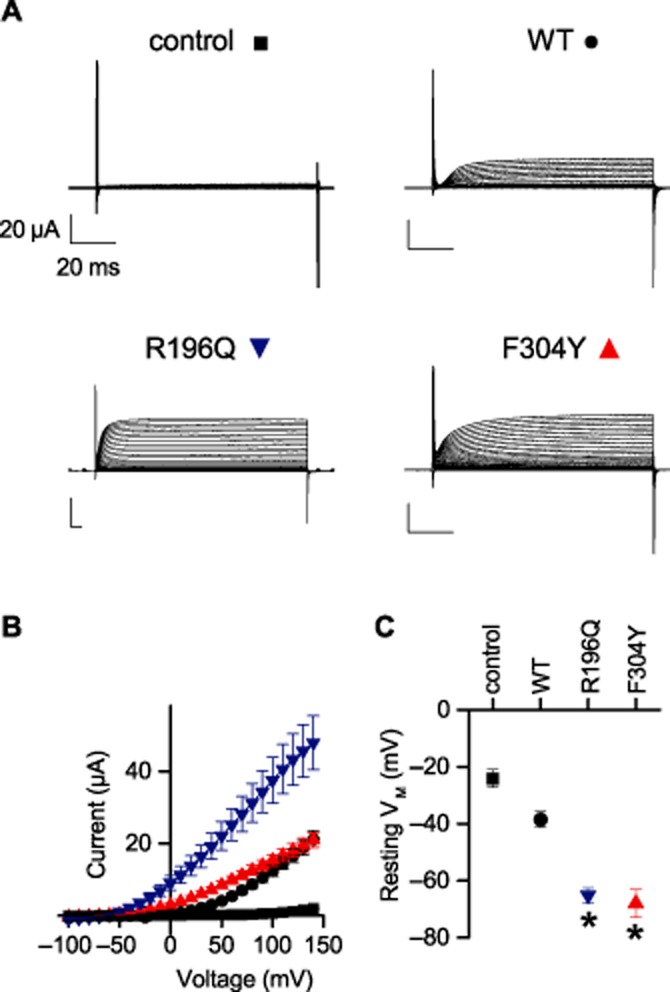
Expression of WT, R196Q and F304Y mSlo3 K^+^ channels in *Xenopus* oocytes. (A) Representative current families recorded by two electrode voltage clamp from oocytes injected with different mSlo3 RNA or with no RNA (control) as indicated. Oocytes were held at −80 mV and 100 ms pulses to potentials between −100 and +140 mV were applied. The dashed line represents the zero-current levels and scale bars represent equivalent current amplitudes and timescales. (B) Mean (± SEM) current–voltage relationships of oocytes expressing WT mSlo3 (WT, *n* = 29), R196Q mSlo3 (*n* = 8) F304Y mSlo3 (*n* = 26), and non-injected oocytes (control, *n* = 12). For symbols used see part (A). (C) Mean (± SEM) resting membrane potential of oocytes in standard Ringer’s solution. *P < 0.0001 compared with control oocytes (Kruskal–Wallis test).

### Effects of KSper inhibitors on WT mSlo3 channels

We next studied the effects of drugs that have previously been shown to inhibit the sperm KSper channel (Navarro *et al*., 2007) on WT mSlo3 currents. Like Ksper (Navarro *et al*., 2007), Slo3 was weakly inhibited by 5 μM mibefridil and 20 mM TEA^+^, but more strongly inhibited by 500 μM quinine and 50 μM clofilium (Figure 2). WT mSlo3 was also strongly inhibited by 2 mM Ba^2+^, an effect that was prevented by raising the extracellular K^+^ concentration to 100 mM (Figure 2). The inhibition by each drug was reversible, although we observed that the amplitude of the current after washing out mibefridil was often larger than the control currents.

**Figure 2:**
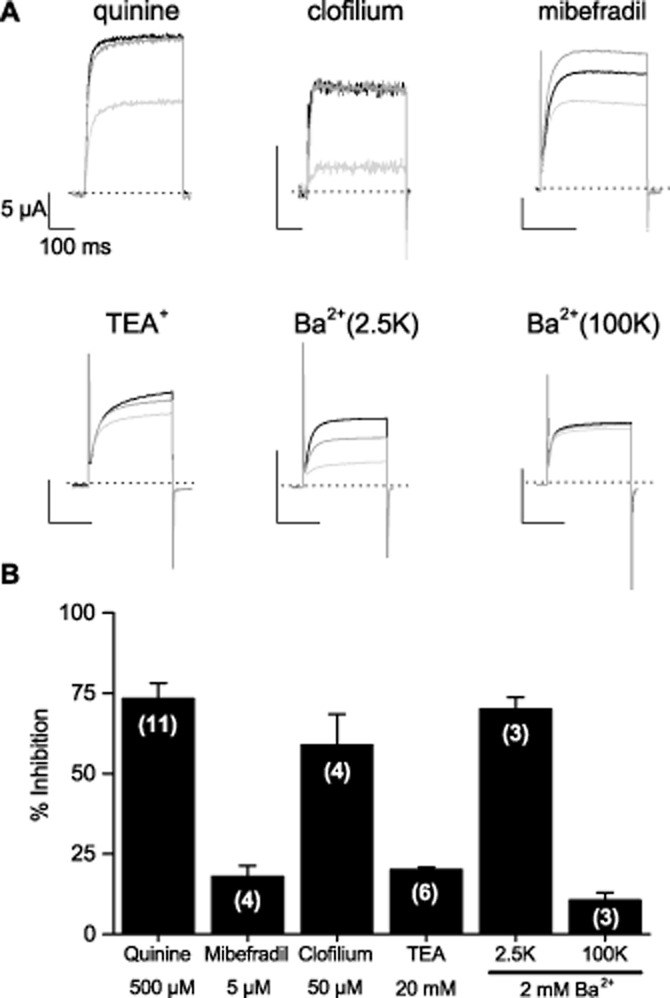
Block of mSlo3 currents by inhibitors of KSper. Oocytes were held at −80 mV and depolarizing pulses to +100 mV were applied. (A) Representative traces with each drug or condition as indicated: black trace, control currents recorded prior to drug application; light grey trace, current in the presence of the inhibitor; dark grey trace, current after washing out the inhibitor for at least 10 min. The dashed line represents the zero-current level and scale bars represent equivalent current amplitudes and timescales. The effects of Ba^2+^ were measured in both the standard solution containing 2.5 mM K^+^ (2.5K) and with a high 100 mM K^+^ solution (100K). (B) Mean percentage inhibition for each drug or condition as indicated (*n* values indicated in parentheses in the bars).

### Block by quinine and quinidine

We studied the effects of quinine and quinidine in more detail. Quinine inhibited WT mSlo3 currents evoked by voltage pulses to +100 mV with an IC_50_ of 169 ± 40 μM and Hill slope of 1.0 ± 0.21 (*n* = 12; Figure 3A and C). F304Y mSlo3 was also blocked by quinine, but with a significantly lower IC_50_ of 15.9 ± 3.31 μM (*n* = 16, *P* < 0.0005; Hill slope of 0.69 ± 0.02). Potency was not significantly altered with the R196Q mSlo3 mutant, which was blocked by quinine with an IC_50_ of 166 ± 27.5 μM and Hill slope of 1.3 ± 0.04 (*n* = 4). The effects of quinidine, a stereoisomer of quinine, have previously been studied on the MC13 Slo1/Slo3 chimera (Tang *et al*., 2010b). Quinidine blocked WT mSlo3 channels with an IC_50_ of 19.9 ± 1.41 μM and Hill slope of 1.15 ± 0.15 (*n* = 7; Figure 3B and D). Again, the potency of inhibition by quinidine was higher for F304Y mSlo3 (IC_50_ of 2.42 ± 0.60 μM, *n* = 9, *P* < 0.005, anova; Hill slope of 0.98 ± 0.12), but lower with R196Q mSlo3 (IC_50_ of 38.4 ± 6.77 μM, *n* = 5, *P* < 0.001, anova; Hill slope of 1.05 ± 0.16). The inhibition of F304Y mSlo by quinidine was observed to have some time dependence (Figure 3B). To gain an insight into the mechanisms by which quinine and quinidine block WT and F304Y mSlo3 channels with altered potencies, we estimated the voltage dependence of the IC_50_ at different voltages. Exemplar currents evoked by voltage ramps and inhibition by quinidine are shown in Supporting Information Figure S2. We found that the F304Y mutation significantly increased the electrical distance, δ, sensed by the blockers from −0.12 ± 0.07 (*n* = 12) to −0.49 ± 0.22 (*n* = 12, *P* < 0.01) for block by quinine (Figure 3E), and from −0.12 ± 0.40 (*n* = 7) to −0.46 ± 0.15 (*n* = 9, *P* < 0.05) for block by quinidine (Figure 3F; two-way anova). The negative values for the electrical distance, δ, represent a positively charged molecule moving into the pore from the intracellular face of the channel.

**Figure 3:**
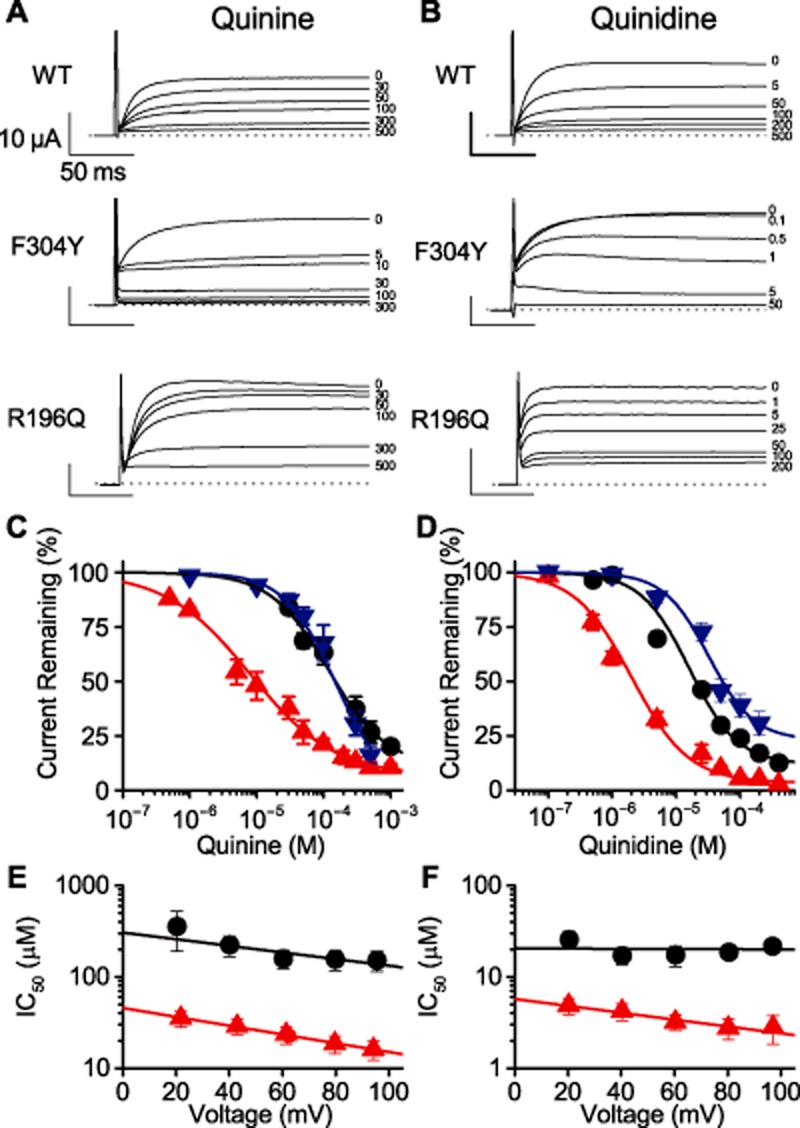
Concentration-dependent inhibition of WT, F304Y and R196Q mSlo3 currents by quinine and quinidine. Representative traces recorded before (0) and in the presence of quinine (A) and quinidine (B) (concentrations in μM as indicated). The dashed line represents the zero-current levels and scale bars represent equivalent current amplitudes and timescales. Mean (± SEM) concentration–inhibition plots for quinine (C) and quinidine (D) inhibition of WT mSlo3, R196Q mSlo3 and F304Y mSlo3 at +100 mV, fitted by the Hill equation provided in the Methods. Apparent voltage-dependence of the IC_50_ for quinine (E) and quinidine (F) of WT and F304Y mSlo3 currents. The data are described and analysed further in the main text. For key to symbols used see Figure 1.

### Inhibition by Ba^2+^ ions

To determine if the F304Y mutation had more wide-ranging effects on the structure of the pore we studied inhibition by Ba^2+^ ions. Concentration–inhibition curves were generated for both WT and F304Y mSlo3 currents evoked by depolarizing steps to +100 mV. Despite the differences in channel activity, the properties of inhibition by Ba^2+^ were similar. The IC_50_ was 646 ± 100 μM (*n* = 6) and 525 ± 97 μM (*n* = 5), with Hill slopes of 0.88 ± 0.11 and 1.05 ± 0.12 for WT and F304Y mSlo3 respectively (Figure 4A and B). Inhibiting concentrations of Ba^2+^ also appeared to slow the activation of the currents upon depolarization (Figure 4A). Voltage dependence of Ba^2+^ inhibition was estimated by measuring the apparent IC_50_ at different voltages, as before. Inhibition of WT and F304Y mSlo3 by Ba^2+^ was not significantly different with δ values of 0.20 ± 0.09 and 0.12 ± 0.05 respectively (Figure 4C).

**Figure 4:**
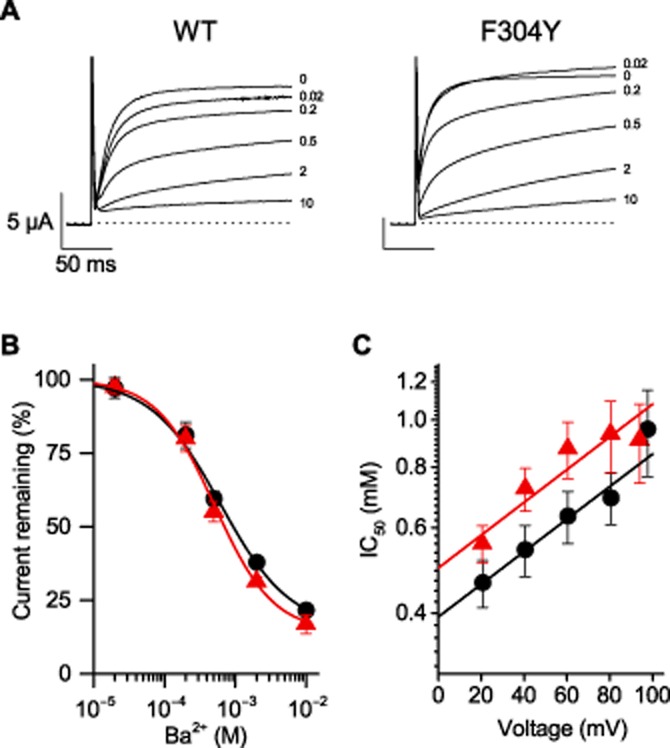
Concentration-dependent inhibition of WT and F304Y mSlo3 currents by Ba^2+^. (A) Representative traces recorded before (0) and in the presence of Ba^2+^ (concentrations in mM as indicated). The dashed line represents the zero-current levels and scale bars represent equivalent current amplitudes and timescales. (B) Mean (± SEM) concentration inhibition plots for WT and F304Y mSlo3 at +100 mV, fitted by the Hill equation provided in the Methods. (C) Apparent voltage-dependence of the IC_50_ for block by Ba^2+^ of WT and F304Y mSlo3-mediated currents. The data are described and analysed further in the main text. For key to symbols used see Figure 1.

### Structural model of mSlo3 and inhibition by quinidine

Both Phyre2 and SWISS-MODEL produced homology models of the mSlo3 transmembrane domain, which were highly similar to each other showing a root mean square deviation of 0.43 Å, giving confidence to the resulting model. Models based on the bacterial cyclic nucleotide-regulated ion channel (PDB: 3BEH) or other K^+^ channel structures showed a common architecture. Docking of quinidine by SwissDock produced a number of predicted mSlo3 binding sites. Most sites were located in the periphery of the K^+^ channel protein at the protein-lipid interface, likely due to the hydrophobic nature of quinidine and, due to the distance from the pore, therefore false-positive results. However, SwissDock also predicted a quinidine-binding site, which involved pore-lining residues F304, I308 and V312. Analysis of the model shows how quinidine is accommodated within a hydrophobic pocket, complementing the hydrophobic character of the inhibitor. Moreover, the replacement of F304 by tyrosine results in the phenolic group being sufficiently close to potentially form a hydrogen bond to the nitrogen on the methoxyquinoline group of quinidine (Figure 5C), which might improve the efficacy of quinidine binding to F304Y mSlo3. Interestingly, SwissDock did not identify any binding sites within the pore for quinidine when F304 was changed to Y304 in the homology model. This may be caused by the bulkier nature of the tyrosine side chain not accommodating the bound inhibitor, since binding site plasticity and side-chain movement is not accounted for in SwissDock.

**Figure 5:**
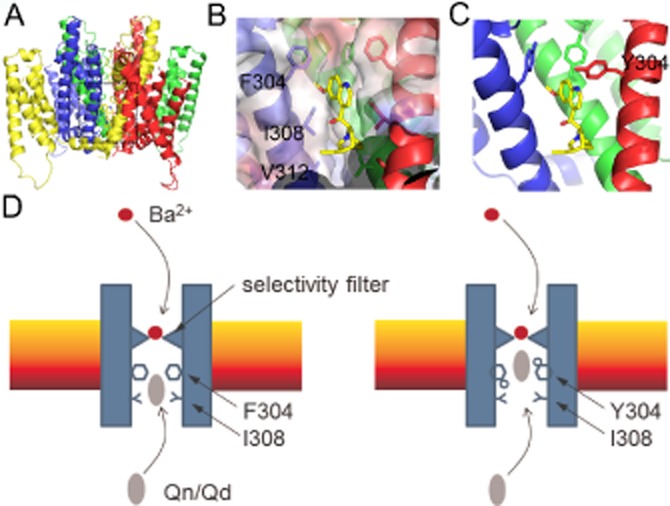
Molecular modelling of mSlo3 and inhibition by quinidine. (A) Homology model of the transmembrane regions S1 to S6 of mSlo3; the structure is shown as a side-on view from the membrane, with the extracellular space above the protein. Each subunit of the tetrameric structure is coloured differently for clarity and only side chains F304, I308 and V312 are shown in stick format. (B) A zoomed-in view of the proposed quinidine-binding site, with subunits labelled blue, red and green, (the fourth subunit has been removed for clarity). The residues which make up the hydrophobic binding pocket (F304, I308 and V312) are shown in stick format and the surface shows the hydrophobic isoelectric character. (C) The same view as in (B) but with tyrosine replacing F304, showing the proximity of the terminal oxygen to the methoxyquinoline group of quinidine where hydrogen bonding could occur. In both (B) and (C), the quinidine molecule is shown in stick format and coloured, yellow, blue and red for carbon, nitrogen and oxygen respectively. (D) Proposed mechanisms for the inhibition induced by Ba^2+^ ions and quinine or quinidine (Qn/Qd): Ba^2+^ enters the pore from the extracellular side and blocks at the selectivity filter; quinine and quinidine cross the membrane and block from the intracellular side at a site involving F304 and I308 (left). In the F304Y mutation (right), the Ba^2+^ block is unaffected, but a deeper binding site is available for quinine and quinidine entering the pore from the intracellular side. Possible mechanisms are discussed in the main text.

## Discussion

We investigated the characteristics of mSlo3 channel inhibition by known blockers by generating mutations that alter open probability. The observation that the R196Q and F304Y mutations both resulted in increased channel activity, particularly at more negative voltages, demonstrates a close structure–function relationship with Slo1 (BK_Ca_, K_Ca_1.1) subunits. The hyperpolarized membrane potentials with R196Q and F304Y mSlo3 expression demonstrate that the K^+^ selectivity of the channel, previously thought to be weak (Schreiber *et al*., 1998), is sufficient to hyperpolarize the membrane to strongly negative potentials when channels are activated. Increased activity at negative potentials *in vivo* is thought to be brought about by co-expression with accessory proteins; candidate accessory proteins are Sloβ4 (Yang *et al*., 2009) and LRRC52 (Yang *et al*., 2011; Zeng *et al*., 2015). We found that the mSlo3 channel has a pharmacological profile similar to that of native mouse KSper channels (Navarro *et al*., 2007) providing further support that mSlo3 underlies, at least in part, this current.

The results with quinine and quinidine indicate that they both block the channel by the same mechanism and site, although with slightly different potency. Quinidine inhibited WT mSlo3 at +100 mV with similar concentrations as those described with the Slo1/Slo3 chimera containing the mSlo3 pore (Tang *et al*., 2010b). The voltage-dependence of inhibition by quinidine was also found to be weak, although no inhibitory effect was observed with voltages more positive than those used in the present study (Tang *et al*., 2010b). The negative slopes that we obtained from the Woodhull plots with block by quinine and quinidine indicate that the drugs enter the mSlo3 channel pore from the intracellular side, presumably by traversing the membrane in the unprotonated state. The efficiency of protonation of the drugs in the cytoplasm, which may be affected by the pH buffering of the oocyte, may contribute to the variability in IC_50_ values. Nonetheless, an interesting effect of the F304Y mSlo3 mutation was its increased sensitivity to inhibition by both quinidine and quinidine compared to WT mSlo3. This observation was initially thought to be as a result of open channel blockade, where the increased open probability of the channels increased the apparent sensitivity to inhibition. However, the results of further experiments provided a better explanation for this observation. Firstly, the F304Y mutation increased the voltage- dependence of block by quinine and quinidine, suggesting that these drugs are able to bind deeper into the F304Y mSlo3 pore. Increased sensitivity to either quinine or quinidine was not observed with the large gain-of-function produced by the R196Q mutation, which rules out increased open probability of this channel as the cause of this effect. The F304Y mutation resulted in a novel binding site where quinine and quinidine were able to block mSlo3 with greater potency than WT mSlo3 channels. The time-dependence of the inhibition of F304 mSlo3 by quinidine may reflect the movement of the drug into the pore induced by the strong depolarization, although an interaction close to the gate at this deeper site cannot be ruled out. The results from the molecular modelling indicate the possibility that this mutation allows an additional hydrogen bond between the tyrosine (Y304) to the methoxyquinoline group of quinidine, which would increase the affinity of the channel pore to quinine or quinidine. An alternative explanation is that the F304Y mutation results in a conformational position of the S6 helix that reveals a higher affinity binding site deeper in the pore. Interestingly, the effect of this mutation is complemented by the findings with HERG (human ether-a-go-go related gene) K^+^ channels, where a structurally analogous, but reverse mutation, Y652F, suppressed the voltage-dependent block by quinidine (Sanchez-Chapula *et al*., 2003). Our molecular models can be further refined through further structural and pharmacological analysis.

The alleviation of the Ba^2+^-induced block of WT mSlo3 observed on raising the extracellular K^+^ concentration is consistent with increased occupancy by K^+^ at a site in the pore analogous to the Slo1 (BK_Ca_} ‘external lock-in’, which prevents Ba^2+^ from entering the pore and binding at a deeper site. The relative locations of K^+^ and Ba^2+^ ions in a K^+^-selective pore have also been demonstrated crystallographically with the Kcsa channel (Jiang and MacKinnon, 2000), and we propose that these corresponding cation binding sites exist in mSlo3. Extracellular K^+^ concentration is therefore a major factor affecting potency of block by extracellular Ba^2+^. Furthermore, the similar results obtained from studies of Ba^2+^ blockade of WT and F304Y mSlo3 demonstrate that this mutation did not affect the selectivity filter. Ba^2+^ blocked both WT and F304Y mSlo3 with similar potency and voltage-dependence and the positive slope of the Woodhull plot reinforces the idea that externally applied Ba^2+^ enters the pore from outside of the cell. The slower current activation in the presence of Ba^2+^ is consistent with the block being alleviated upon depolarization and further supports the idea that this ion can block the channel whilst it is closed. The negative resting membrane potentials that were obtained by expressing either R196Q or F304Y mSlo3 indicate that the latter mutation had no appreciable effect on the ionic selectivity of the mSlo3 pore.

Finally, our results suggest that the location of the activation gate in Slo3 channels may be similar to that of the closely related Slo1 (BK_Ca_) channel. We conclude that inhibition of mSlo3 by barium and quinine/quinidine are not dependent on the channel being in the open or closed state. This indicates that the activation gate lies between the Ba^2+^ and quinine/quinidine-binding sites in WT mSlo3. Structurally, this points to a region deep within the pore between the selectivity filter and F304, and not at an S6 helix bundle at the intracellular face. This idea corresponds well with the conclusions of previous studies on BK_Ca_ channels in that an S6 helix bundle is unlikely to form a gate to permeating ions (Wilkens and Aldrich, 2006; Zhou *et al*., 2011), and that gating may involve side-chain reorientation of S6 residues deep within the pore and close to the selectivity filter (Chen *et al*., 2014).

To conclude, the extracellular K^+^ concentration negatively influences the potency of Ba^2+^ in blocking Slo3 K^+^ channels, and probably, therefore, KSper. We propose that quinine and quinidine inhibit Slo3 channels by binding to hydrophobic side chains found at the intracellular region of the channel pore. We also described two mutations in mSlo3 that increased channel activity and demonstrate a close structure–function relationship to Slo1 (BK_Ca_; K_Ca_1.1) channels. It is expected that pharmacological modulation of Slo3 K^+^ channels will affect the function of spermatozoa and this ion channel presents a novel therapeutic target in the treatment or control of male fertility.

## Abbreviations

KSper: sperm potassium conductance
mSlo3: mouse Slo3 (K_Ca_5.1) channel
WT: wild type

## Supporting Information

**Figure S1** A–C. Currents recorded from oocytes expressing WT and mutant mSlo3 as indicated following perfusion of bath solution containing 98 mM KCl and no sodium. The holding potential was −80 mV and pulses were applied in 10 mV steps to up to +140 mV. The scale bars apply to each panel and the zero-current levels are indicated by the dashed lines. Mean ± SEM. (*n* = 5 or 6) current- (D) and conductance-voltage (E) relationships from oocytes expressing WT (●), F304Y (▲) and R196Q (▼) mSlo3. F Relationship between reversal potentials measured and bathing [K+], where [Na+] = 98 mM − [K+]. **Figure S2** Block by quinidine of currents evoked by a voltage ramp protocol from oocytes expressing WT (A), F304Y (B), and R196Q (C) mSlo3. (D) The time-course of the voltage ramp. The concentration of quinidine, in μM, is shown to the right of the current trace, with *0* the control current prior to perfusing quinidine.
