# Supplementary material for: Mechanism of inhibition of mouse Slo3 (KCa5.1) potassium channels by quinine, quinidine and barium
Source: Br J Pharmacol. 2015 Jul 14;172(17):4355–63. doi: 10.1111/bph.13214 (PMC4556473; doi:10.1111/bph.13214)
Supplement: Supplementary file 1 [file bph0172-4355-sd1.pdf]

Mechanism of inhibition of mouse Slo3 (KCa5.1) potassium channels by quinine, quinidine, and barium.

D C Wrighton, S P Muench, and J D Lippiat

Supplementary Data

Further characterisation of the effects of the mutations F304Y and R196Q on mSlo3 function were carried out by recording currents in ND-96 solution (96 mM NaCl, 2 mM KCl, 1.8 mM $CaCl_2$, 2 mM $MgCl_2$, 5 mM HEPES, pH7.5) and in solutions where NaCl was replaced by equimolar KCl. By measuring current-voltage-relationships in 98 mM K^+ solution (no Na^+) (Supplementary Figure 1A-D) the reversal potential of F304Y and R196Q mSlo3 currents could be determined and channel activity was expressed as conductance (Supplementary Figure 1E). We were unable to measure the reversal potential of WT mSlo3 as there was negligible current at negative voltages, and so this was constrained to the mean reversal potential obtained from the mutant mSlo3 channels. The mean ± s.e.m. half-maximal activation voltages, obtained by fitting Boltzman functions to individual conductance-voltage (G-V) relationships, were 75.7 ± 5.7 mV (n=6), -4.7 ± 6.3 mV (n= 6), and -139.4 ± 24.0 (n=5) for WT, F304Y, and R196Q mSlo3, respectively. The plots illustrate that WT mSlo3 does not reach full activity in this voltage range, whilst the mutant channels are near-full activity at +100 mV.

We also investigated the ion selectivity of mutant mSlo3 channels by measuring reversal potentials in different extracellular ($[K^+]_o$) concentrations. Owing to the high activation threshold and rapid deactivation kinetics of WT mSlo, we were unable to resolve the reversal potential from endogenous *Xenopus* oocyte currents at negative potentials, neither from tail currents following step voltage pulses nor from voltage ramps. Data from oocytes expressing F304Y or R196Q mSlo3 (Supplementary Figure 1F) were fitted with the function $E_{rev} = (RT/zF).\ln(([K^+]_o+\alpha[Na^+]_o)/[[K^+]_i+\alpha[Na^+]_i])$, where α is the permeability ratio P_{Na}/P_K, $[Na^+]_o$ = 98 mM – $[K^+]_o$; $[K^+]_i$ and $[Na^+]_i$ were constrained to 109.5 mM and 10.1 mM, respectively (Costa *et al.*, 1989). F304Y and R196Q mSlo3 currents gave mean (± s.e.m.) P_{Na}/P_K permeability ratios of 0.024 ± 0.007 (n=5) and 0.011 ± 0.002 (n=6), respectively.

In the present study we describe the assessment of the dependence of drug block on membrane potential. For this, we used a 1500 ms voltage ramp that enables the degree of current inhibition to be determined at different voltages across the sweep. Exemplar currents are shown in Supplementary Figure 2 where inhibitions by quinidine of currents evoked by voltage ramps were recorded from oocytes expressing each of the three mSlo3 variant.

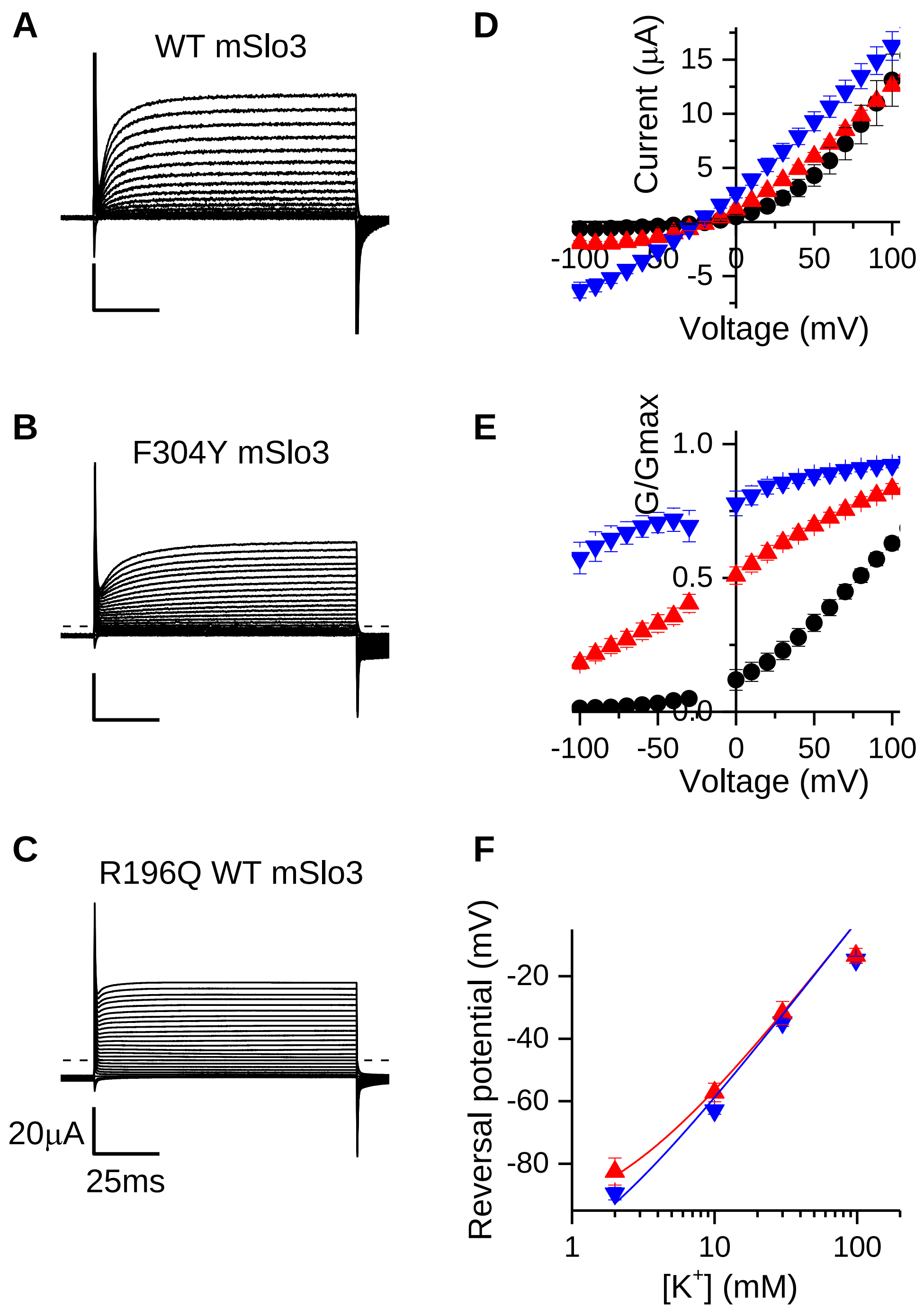

Supplementary Figure 1

A-C Currents recorded from oocytes expressing WT and mutant mSlo3 as indicated following perfusion of bath solution containing 98 mM KCl and no sodium. The holding potential was -80 mV and pulses were applied in 10 mV steps to up to +140 mV. The scale bars apply to each panel and the zero current levels are indicated by the dashed lines. Mean ± s.e.m. (n= 5 or 6) current- (**D**) and conductance-voltage (**E**) relationships from oocytes expressing WT (●), F304Y (▲), and R196Q (▼) mSlo3. F Relationship between reversal potentials measured and bathing $[K^+]$, where $[Na^+]$ = 98 mM – $[K^+]$.

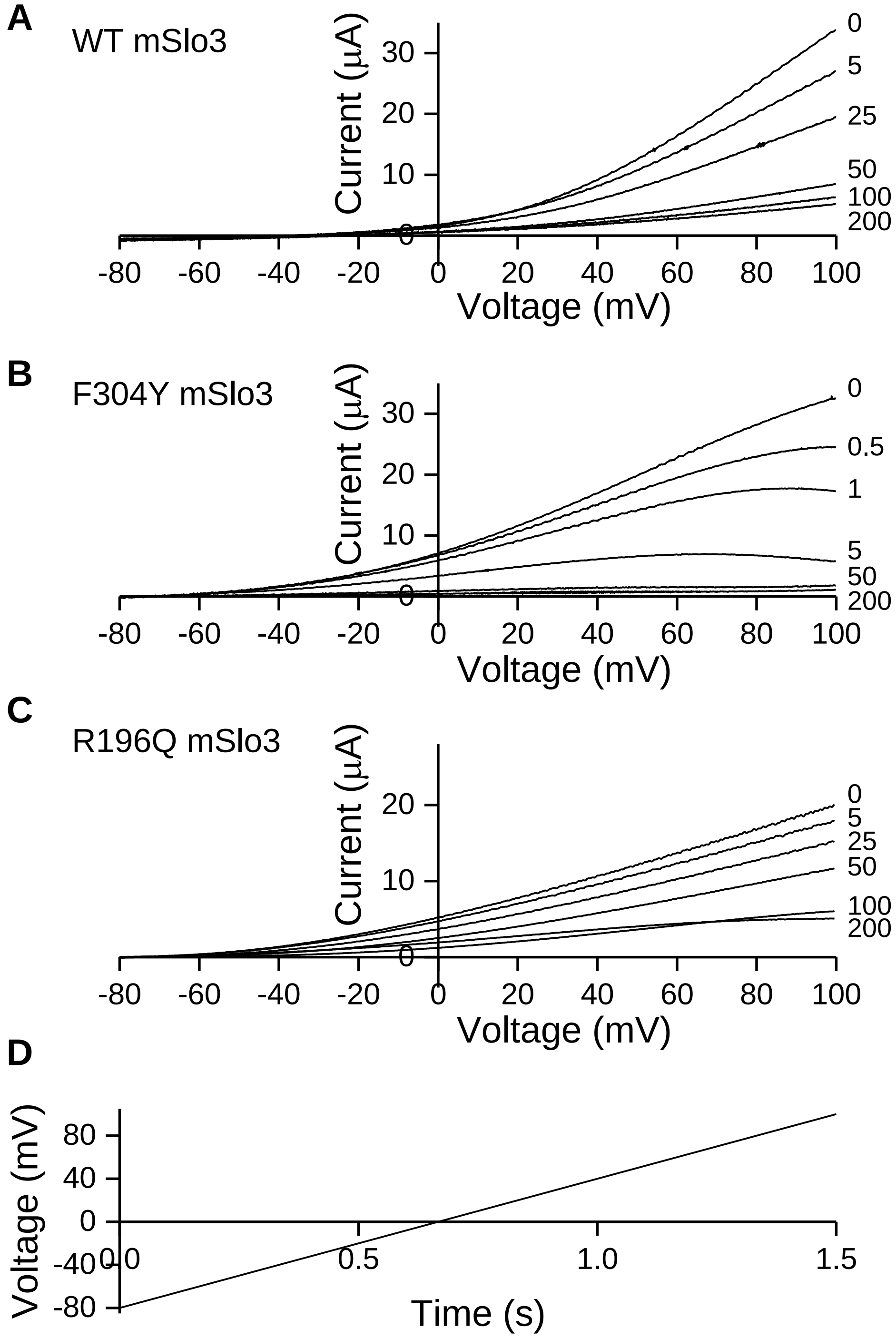

Supplementary Figure 2

Block by quinidine of currents evoked by a voltage ramp protocol from oocytes expressing WT (**A**), F304Y (**B**), and R196Q (**C**) mSlo3. **D** The time-course of the voltage ramp. The concentration of quinidine, in μM, is shown to the right of the current trace, with *0* the control current prior to perfusing quinidine.
